# Biomechanical evaluation of reinsertion and revision screws in the subaxial cervical vertebrae

**DOI:** 10.1186/s12891-023-07158-3

**Published:** 2024-05-21

**Authors:** Wei-xin Dong, Yong Hu, Ou-jie Lai, Zhen-shan Yuan, Xiao-yang Sun

**Affiliations:** Department of Spinal Surgery, Ningbo No.6 Hospital, 1059 East Zhongshan Road, Ningbo, Zhejiang 315040 People’s Republic of China

**Keywords:** Cervical, Vertebrae screw, Reinsertion, Insertional torque, Pullout strength, Biomechanical

## Abstract

**Background:**

This study aimed to evaluate the biomechanical effects of reinserted or revised subaxial cervical vertebral screws.

**Methods:**

The first part aimed to gauge the maximum insertional torque (MIT) of 30 subaxial cervical vertebrae outfitted with 4.0-mm titanium screws. A reinsertion group was created wherein a screw was wholly removed and replaced along the same trajectory to test its maximum pullout strength (MPOS). A control group was also implemented. The second part involved implanting 4.0-mm titanium screws into 20 subaxial cervical vertebrae, testing them to failure, and then reinserting 4.5-mm revision screws along the same path to determine and compare the MIT and MPOS between the test and revision groups.

**Results:**

Part I findings: No significant difference was observed in the initial insertion’s maximum insertion torque (MIT) and maximum pull-out strength (MPOS) between the control and reinsertion groups. However, the MIT of the reinsertion group was substantially decreased compared to the first insertion. Moderate to high correlations were observed between the MIT and MPOS in both groups, as well as between the MIT of the first and second screw in the reinsertion group. Part II, the MIT and MPOS of the screw in the test group showed a strong correlation, while a modest correlation was observed for the revision screw used in failed cervical vertebrae screw. Additionally, the MPOS of the screw in the test group was significantly higher than that of the revision screw group.

**Conclusion:**

This study suggests that reinsertion of subaxial cervical vertebrae screws along the same trajectory is a viable option that does not significantly affect fixation stability. However, the use of 4.5-mm revision screws is inadequate for failed fixation cases with 4.0-mm cervical vertebral screws.

**Supplementary Information:**

The online version contains supplementary material available at 10.1186/s12891-023-07158-3.

## Background

While the use of anterior cervical plate (ACP) fixation technology is prevalent in clinical practice [1–3], there remains a paucity of research addressing the biomechanical stability of the cervical vertebrae screw (CVS), which plays a crucial role as the anchoring point for the ACP. Cervical vertebrae screws are frequently employed in spinal surgeries [1–3], and screw reinsertion may be required in a variety of circumstances. Despite this, there is a lack of biomechanical investigations utilizing maximum pull-out strength (MPOS) testing on human cadaveric models to assess the immediate fixation strength following screw reinsertion. Additionally, there is a dearth of literature on the efficacy of 4.5-mm revision screws in providing adequate biomechanical stability in cases of failed 4.0-mm screw fixation. Consequently, the present study endeavors to accomplish two aims: first, to assess the biomechanical implications of cervical vertebrae screw reinsertion using the prior trajectory, and second, to determine the immediate fixation strength of 4.5-mm revision screws for failed cervical vertebrae screw fixation.

## Materials and methods

### Specimen Preparation

**Fig. 1:**
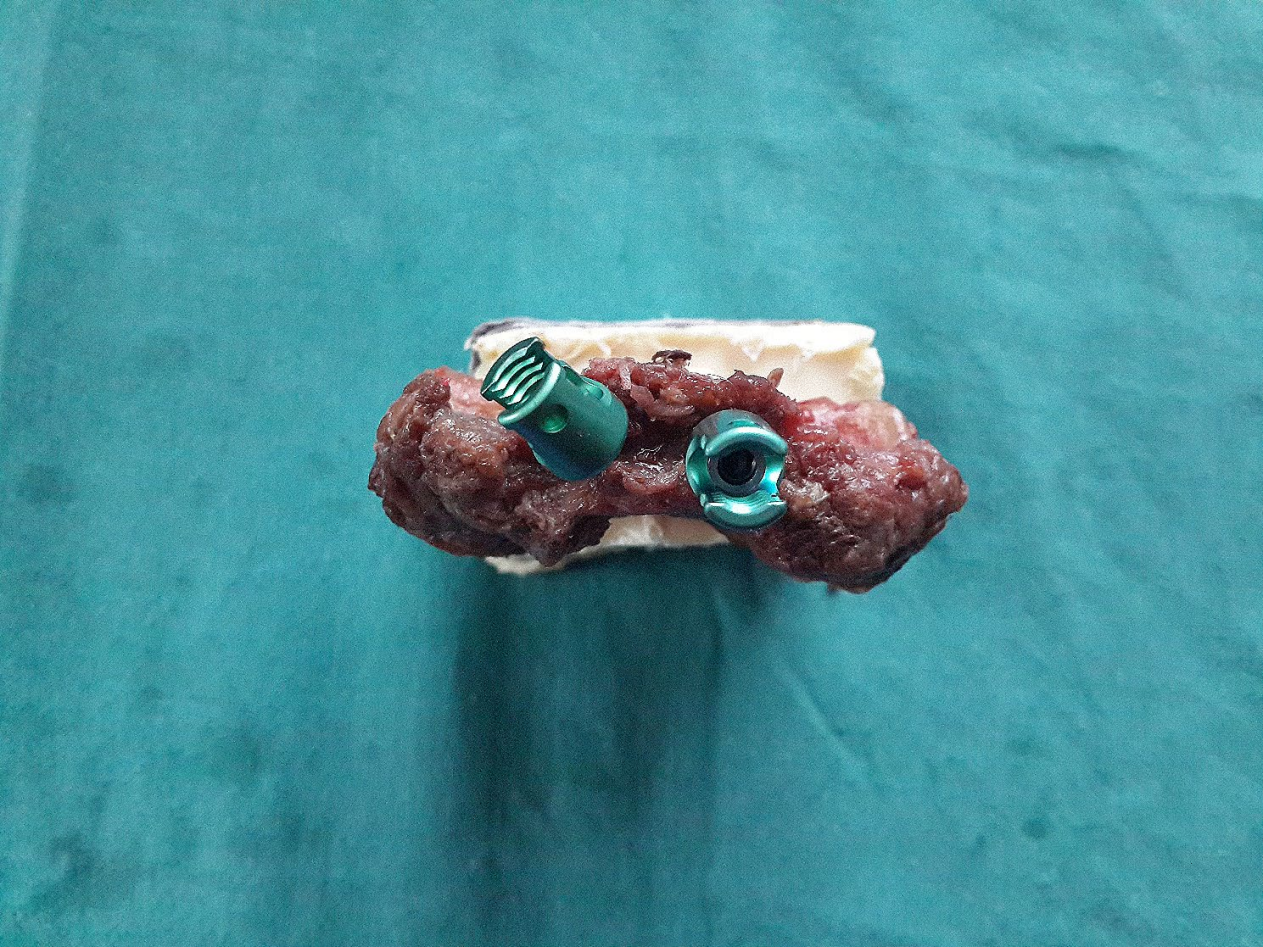
Shows the cervical laminae securely attached to polymethyl methacrylate with cervical vertebral screws inserted

Part I involved the evaluation of biomechanics after reinsertion using a previous trajectory on 30 subaxial cervical vertebrae (C3-7) harvested from human cadaveric spines. The study cohort comprised three males and three females, aged 51 to 84 years with a mean age of 63.7 years. For Part II, 20 subaxial cervical vertebrae (C3-7) were harvested from human cadaveric spines to test the biomechanics of revision screw fixation for failed subaxial cervical vertebrae screw fixation. The cohort included two males and two females, aged 53 to 72 years with a mean age of 60.7 years. Each specimen yielded five cervical vertebrae (C3, C4, C5, C6, C7). Prior to experimentation, the soft tissues, such as muscles, attached to the cervical vertebrae were removed, leaving the bone components of the C3, C4, C5, C6, and C7 vertebrae intact. The cervical spinous process and vertebral plate were then embedded in the embedding box using polymethylmethacrylate (Fig. 1). All ten cervical specimens were obtained from individuals who had died accidentally. Prior to the study, the specimens underwent fluoroscopic screening to ensure the absence of any significant anatomical abnormalities, such as fractures, deformities, dysplasia, pars defects, or congenital anomalies.

### Biomechanical testing

**Fig. 2:**
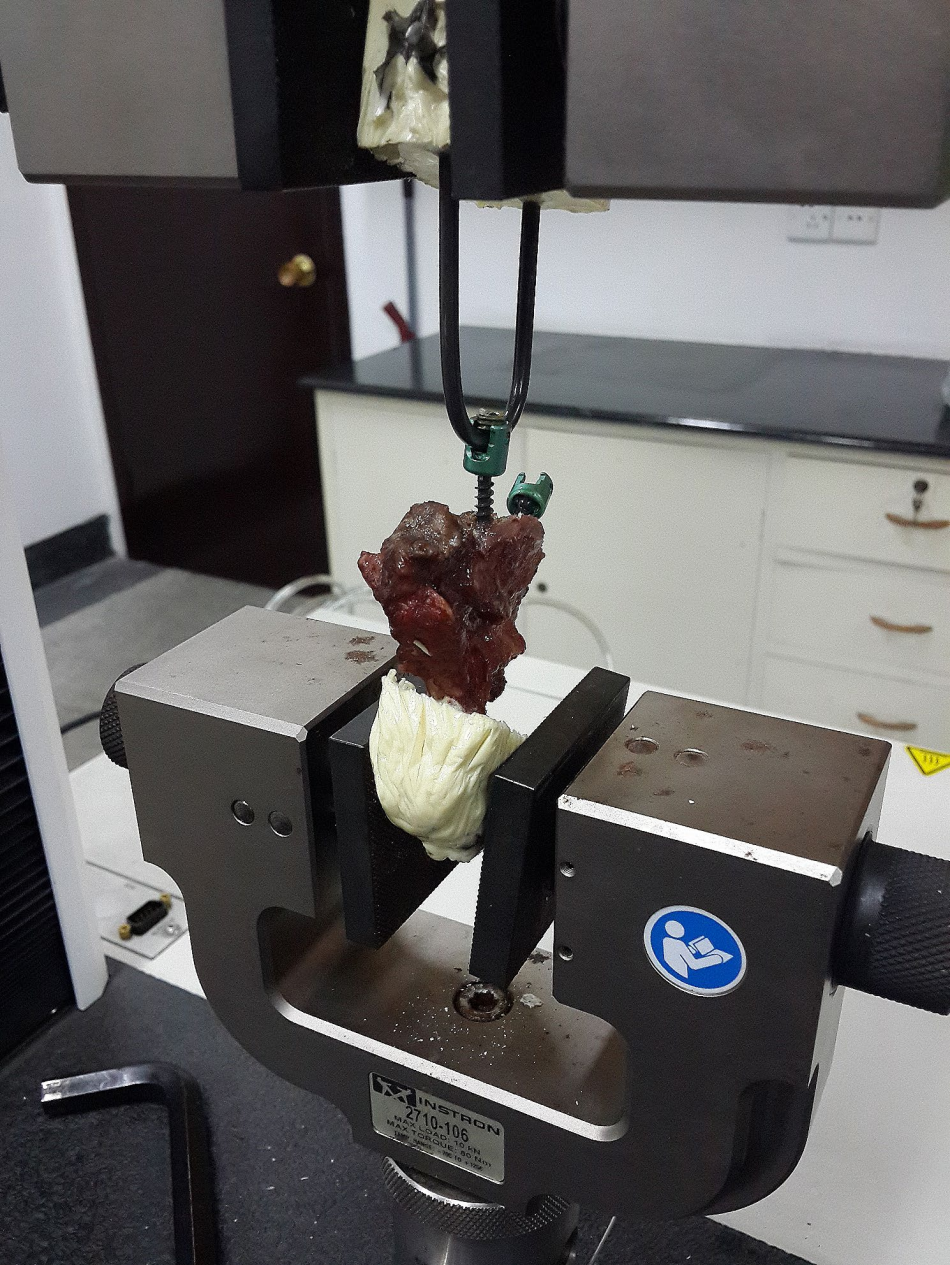
Portrays the experimental configuration, whereby each specimen is individually secured in a pot and aligned with the cervical vertebral screw axis utilizing the Instron 8874 machine (Instron, Canton, Massachusetts)

Part I (n = 30): To assess the biomechanical properties of reinserted cervical vertebrae screws, we conducted an evaluation of the Maximum Insertion Torque (MIT) on both sides of the vertebral body following the placement of 4.0-mm cervical vertebrae screws on both the left and right sides. In this experiment, the insertion depth of the screws (Sanyou, Shanghai, China) is 14 mm. The torque measurement tool employed N10DPSK, Ai Gu, Dongguan, China. One side of the vertebral screw was randomly selected for reinsertion using the previous trajectory (reinsertion group), while the other side served as the control screw (control group). Screws were inserted perpendicular to the surface of the cervical vertebral body within the axial plane, reaching the same depth, and affixed through single cortical fixation. Part II (n = 20): After inserting 4.0-mm diameter screws in the cervical vertebrae, the MPOS test was conducted. After the failure of the 4.0 mm screws, 4.5-mm diameter revision screws were placed in the prior trajectory. The MITs were then recorded, and the angle of the fixed vertebral body was adjusted to match the direction of the screw. The MPOS test was conducted at a displacement rate of 2 mm/min along the cervical vertebral screw’s direction, and the depth of the reinsertion or revision screw was the same as that of the first insertion. MIT measurements were carried out using a torque screwdriver during screw insertion, while MPOS testing was performed using an Instron 8874 biomechanical testing equipment with the serial number is 3366 (Instron, Canton, Massachusetts) (Fig. 2).

### Statistical analysis

Part I (n = 30): The present study utilized SPSS 21.0 statistical software to determine the mean and standard deviation of the maximum insertion torque (MIT) and the maximum pullout strength (MPOS). The paired-samples t-test was employed to examine the statistical significance of differences in MIT or MPOS between the right and left sides at the initial insertion. Additionally, the paired-samples t-test was utilized to investigate any differences in MIT or MPOS between the screw’s first insertion and subsequent reinsertion using a previous trajectory. To assess the correlation between the MIT and MPOS values of the screw’s first insertion and the reinsertion utilizing a previous trajectory, the Spearman correlation analysis was employed. Part II (n = 20): The current study also compared the MIT and MPOS values for the cervical vertebrae screw that was first inserted to those of the revision screw for the failed cervical vertebrae screw. Furthermore, the Spearman correlation analysis was utilized to evaluate the correlation between the MIT and MPOS values of the screw (first 4.0 mm insertions) in the test group and the revision screw (4.5 mm reinsertion) for the failed cervical vertebrae screw. Statistical significance was considered at *P* < 0.01. The correlation coefficient values were categorized as low (≤ 0.49), moderate (0.50–0.69), and high (≥ 0.70), following previous literature.

## Results

### Cervical vertebral screw MIT analysis

In Part I (n = 30) of the study, the mean maximum insertion torque (MIT) for the cervical vertebral screw during its first insertion in the reinsertion group was 2.57 ± 0.81(kgf.cm), which was not statistically different from the control group’s MIT of 2.66 ± 0.85(kgf.cm) (*P* > 0.05). However, the MIT of the screw reinsertion in the reinsertion group (1.53 ± 0.69(kgf.cm)) was significantly lower than that of the control group (*P* < 0.01). In Part II (n = 20), the MIT of the cervical vertebral screw during its first insertion (2.63 ± 0.92(kgf.cm)) was significantly higher than that of the revision screw for failed cervical vertebral screw fixation (1.56 ± 0.38) kgf.cm, with a statistically significant difference (*P* < 0.01).

### Cervical vertebral screw MPOS analysis

In Part I (n = 30), the mean maximum pull-out strength (MPOS) of the cervical vertebral screw in the control group was (435.89 ± 156.99) N, and the MPOS of the screw reinsertion in the reinsertion group (410.79 ± 145.09(N)) showed no significant difference (*P* > 0.05). In Part II (n = 20), the MPOS of the cervical vertebral screw in the test group (408.66 ± 151.87(N)) was significantly higher than that of the screw revision group (187.158 ± 85.27(N)) with a statistically significant difference (*P* < 0.01) (Table 1).

**Table 1:** Comparison of MPOS and MIT results among different groups

	Min. Value	Max. Value	Mean	Std. Deviation	
MPOS of screws in control group (n = 30)	139.82	797.96	435.8992	156.99961	P1 = 0.211
MPOS of reinserted screws in reinsertion group (n = 30)	187.58	825.81	410.7913	145.08824	
MIT of screws in control group (n = 30)	1.09	4.13	2.6564	0.84926	P2 = 0.111
MIT of initially inserted screws in reinsertion group (n = 30)	1.00	4.01	2.5678	0.80589	
MIT of reinserted screws in reinsertion group(compared with initially inserted) (n = 30)	0.44	3.15	1.5267	0.68539	P3 = 0.000
MPOS of screws in test group (n = 20)	181.5840	755.4360	408.657300	151.8699985	P4 = 0.000
MPOS of revision screws (n = 20)	47.4720	380.8800	187.155600	85.2669656	
MIT of screws in test group (n = 20)	1.0450	4.1800	2.637800	0.9224666	P5 = 0.000
MIT of revision screws (n = 20)	0.8360	2.2990	1.564888	0.3752815	

### Correlation between cervical vertebral screw MPOS and MIT

In Part I (n = 30), the correlation between the MIT and MPOS of the cervical vertebral screw was high (r = 0.794, *P* = 0.000) in the control group. A high correlation was also found between the MIT of the screw inserted initially and the MPOS in the reinsertion group (r = 0.761, *P* = 0.000). The MIT of the screw reinsertion and MPOS in the reinsertion group had a moderate correlation (r = 0.547, *P* = 0.002). In the reinsertion group, the correlation between the MIT of the cervical vertebral screw during its first insertion and the MIT of the screw reinsertion was moderate (r = 0.623, *P* = 0.000) (Table 2). In Part II (n = 20), the correlation between the MIT and MPOS of the cervical vertebral screw in the test group was high (r = 0.824, *P* = 0.000), while the correlation between the MIT and MPOS of the revision screw for failed cervical vertebral screw fixation was modest (r = 0.633, *P* = 0.000) (Table 2).

**Table 2:** Correlation between screw insertion torque and pullout strength in different groups

	*r*	*P*
Correlation between MIT and MPOS of the screw in control group (n = 30)	*r* = 0.794	*P* = 0.000
Correlation between MIT with the screw first insertion and MPOS in reinsertion group (n = 30)	*r* = 0.761	*P* = 0.000
Correlation between MIT of the screw reinsertion and MPOS in reinsertion group (n = 30)	*r* = 0.547	*P* = 0.002
Correlation between MIT of the screw first insertion and MIT of the screw reinsertion in reinsertion group (n = 30)	*r* = 0.623	*P* = 0.000
Correlation between MIT and MPOS of the screw in test group (n = 20)	*r* = 0.824	*P* = 0.000
Correlation between MIT and MPOS of the revision screw for failed cervical vertebrae screw (n = 20)	*r* = 0. 633	*P* = 0.000

## Discussion

The anterior cervical plate (ACP) fixation technology has been widely adopted in clinical practice [1–3]. However, there is limited research on the biomechanical stability of the cervical vertebrae screw (CVS), which serves as the anchor point for the anterior cervical plate. As the use of ACP fixation technology increases, cervical vertebrae screw reinsertion using a previous trajectory is becoming more common [4–6], yet the effect of CVS reinsertion on the maximum insertion torque (MIT) and mean maximum pull-out strength (MPOS) has received little attention in the literature. Furthermore, the biomechanics of 4.5 mm diameter CVS for failed screws has not been extensively studied. This study aimed to evaluate the effect of CVS reinsertion using a previous trajectory on MPOS and MIT using biomechanical experiments. The study also aimed to determine if CVS with a diameter of 4.5 mm can provide sufficient biomechanical stability after screw failure. The MPOS, a critical parameter indicating screw stability in clinical and scientific contexts, this might be better stated to indicate that pull out strength was quantitatively assessed under controlled conditions that controlled for the influence of screw outer diameter, feed depth, thread pitch, bone mineral density (BMD), and screw shape on the results. To this end, we measured the two parameters symmetrically on opposite sides of the same vertebrae [7–9].

While it is a goal of surgeons to prevent the need for reinsertion of cervical vertebrae screws (CVS), this may not always be feasible in practice [10–12]. The most common reasons for revision surgery and replacement of CVS are as follows: (1) the presence of a hematoma that causes spinal cord compression following anterior cervical spine surgery, necessitating revision surgery to remove the hematoma and alleviate the compression; (2) incomplete intraoperative decompression resulting in nerve root or residual spinal cord compression, requiring subsequent surgery to achieve complete decompression; and (3) the emergence of additional pressure-causing factors, such as adjacent segment degeneration after anterior fusion, requiring revision surgery to relieve compression or prolong the treated segment. In such cases, complete removal of the CVS is necessary before reinsertion. During revision surgery, surgeons may have concerns about the impact of screw reinsertion on the rigidity of the CVS when utilizing the previous trajectory. However, our research has demonstrated that subsequent surgery with the original screw in the same trajectory can be performed without significant reduction in biomechanical rigidity, provided that the CVS trajectory is not disturbed.

Cervical vertebrae screw (CVS) fixation failure is a common issue, often resulting from compromised screw trajectory [13–15]. Factors that can compromise screw trajectory include: (1) osteoporosis-induced bone loss, bone thinning, and fragile bone trabeculae leading to high bone brittleness and weak screw fixation; (2) suboptimal surgical technique, such as inexperience, cervical lordosis, or poor fracture reduction, which can lead to CVS stress and eventual failure; (3) inappropriate postoperative exercise, unprotected external fixation, and noncompliance with recommended functional exercises, which can exacerbate internal fixation site damage; and (4) trauma after discharge from the hospital, leading to further internal fixation site damage and screw failure. In such cases, physicians may elect to replace 4.0 mm cervical vertebral screws with 4.5 mm diameter screws. However, the biomechanical stability of the larger diameter revision screws remains uncertain following screw failure. Our investigation has revealed that a 4.5 mm diameter revision screw does not offer sufficient biomechanical stability if the cervical vertebral screw trajectory is disrupted. Thus, it is crucial to take appropriate measures to prevent screw failure and ensure proper CVS trajectory during initial fixation to mitigate the need for revision surgery.

## Conclusion

Reinserting cervical screws along the same trajectory can prevent fixation failure and maintain stability, despite reduced insertion torque. But, using 4.5-mm revision screws for 4.0-mm screw fixation failure may not provide enough pullout strength. Surgeons should consider individual patient conditions and anatomy before choosing treatment options.

## Limitation

Considering the constraints associated with anterior cervical screws, which may not offer optimal clamping, we chose polyaxial screws over cervical anterior vertebral body screws, introducing a notable limitation. The screws are inserted as vertically as possible to the anterior surface of the cervical vertebral body. However, this process may introduce some deviation. Given the variability in screw designs this argument may also be an extrapolation. We acknowledge that the restricted sample size may impact the generalizability of our findings and is indeed a limitation of the study.

### Electronic supplementary material

Below is the link to the electronic supplementary material.

Supplementary Material 1

Supplementary Material 2

## Data Availability

All data generated or analysed during this study are included in this published article.

## Abbreviations

MPOS: Maximum pullout strength
MIT: Maximum insertional torque
ACP: Anterior cervical plate
CVS: Cervical vertebral screw

## References

[1] Sarker SK, Islam SS, Mahmud CI, Islam MA, Rahman MM, Rahman SI (2022). Anterior cervical discectomy and Fusion (ACDF) with stabilization by cervical locking plate and screw in traumatic sub-axial incomplete cervical spine injury: early experience. Int J Orthop Sci.

[2] Salzmann SN, Okano I, Miller CO, Chiapparelli E, Reisener MJ, Winter F (2020). Regional bone mineral density differences measured by quantitative computed tomography in patients undergoing anterior cervical spine Surgery. Spine J.

[3] Song KJ, Choi BW, Ham DH, Kim HJ (2020). Prognosis of hardware-related problems in anterior cervical discectomy and fusion with cage and plate constructs. World Neurosurg.

[4] Okawa A, Sakai K, Hirai T, Kato T, Tomizawa S (2011). Risk factors for early reconstruction failure of multilevel cervical corpectomy with dynamic plate fixation. Spine.

[5] Mullins J, Pojskić M, Boop FA, Arnautović KI (2018). Retrospective single-surgeon study of 1123 consecutive cases of anterior cervical discectomy and fusion: a comparison of clinical outcome parameters, complication rates, and costs between outpatient and inpatient Surgery groups, with a literature review. J Neurosurg.

[6] Lovasik BP, Holland CM, Howard BM, Baum GR, Rodts GE, Refai D (2017). Anterior cervical discectomy and fusion: comparison of fusion, dysphagia, and complication rates between recombinant human bone morphogenetic protein-2 and beta-tricalcium phosphate. World Neurosurg.

[7] Anderson KD, Ko FC, Virdi AS, Sumner DR, Rosset RD (2020). Biomechanics of implant fixation in osteoporotic bone. Curr Osteoporos Rep.

[8] Varghese V, Krishnan V, Kumar GS (2019). Comparison of pullout strength of pedicle screws following revision using larger diameter screws. Med Eng Phys.

[9] Hsieh MK, Li YD, Hsu YJ, Tsai TT, Lai PL, Lee DM (2022). Novel dual-threaded pedicle screws provide fixation Stability that is comparable to that of traditional screws with relative bone preservation: an in Vitro Biomechanical Study. Appl Sci.

[10] Bovonratwet P, Fu MC, Tyagi V, Bohl DD, Ondeck NT, Albert TJ (2019). Incidence, risk factors, and clinical implications of postoperative hematoma requiring reoperation following anterior cervical discectomy and fusion. Spine.

[11] Taghvaei M, Meybodi KT, Zeinalizadeh M (2018). Ligamentum flavum buckling causing immediate post-operative neurological deterioration after an anterior cervical discectomy: case report. Turk Neurosurg.

[12] Alhashash M, Shousha M, Boehm H (2018). Adjacent segment Disease after cervical spine fusion: evaluation of a 70 patient long-term follow-up. Spine.

[13] Shen FH, Samartzis D (2008). Careful follow-up after successful Surgery: postoperative spondylolisthesis after anterior cervical corpectomy and fusion with instrumentation. Surg Neurol.

[14] Dorai Z, Morgan H, Coimbra C (2003). Titanium cage reconstruction after cervical corpectomy. J Neurosurgery: Spine.

[15] Ponnusamy KE, Iyer S, Gupta G, Khanna AJ (2011). Instrumentation of the osteoporotic spine: biomechanical and clinical considerations. Spine J.

